# A Stratified Analysis of the Associations of Hearing Loss and Allergic Rhinitis with Attention Deficit Hyperactivity Disorder in Children: A Population-Based Cross-Sectional Study in Korea

**DOI:** 10.3390/ijerph22091422

**Published:** 2025-09-12

**Authors:** Bo Ram Yang, Bong Jik Kim

**Affiliations:** 1 Chungnam National University College of Pharmacy, Daejeon 34134, Republic of Korea; br.yang@cnu.ac.kr; 2 Department of Bio-AI Convergence, Chungnam National University, Daejeon 34134, Republic of Korea; 3 Department of Otolaryngology-Head and Neck Surgery, Chungnam National University College of Medicine, Chungnam National University Sejong Hospital, Sejong 30099, Republic of Korea; 4 Brain Research Institute, Chungnam National University College of Medicine, Daejeon 35015, Republic of Korea; 5 Department of Otorhinolaryngology-Head and Neck Surgery, Chungnam National University Sejong Hospital, Sejong 30099, Republic of Korea

**Keywords:** attention deficit hyperactivity disorder (ADHD), hearing loss, allergic rhinitis, big data, association

## Abstract

We conducted a cross-sectional, population-based study to investigate the association between hearing loss and allergic rhinitis with attention deficit hyperactivity disorder (ADHD) using pediatric patient samples from the Korea Health Insurance Review and Assessment Service database (2009–2018). Baseline demographics and the relationships of both conditions with ADHD were analyzed. Both hearing loss and allergic rhinitis were significantly associated with ADHD, and patients with both hearing loss and allergic rhinitis showed a stronger association with ADHD compared to patients with either hearing loss or allergic rhinitis alone. The association of hearing loss with ADHD appeared to be stronger in girls than in boys. Our study revisited the link between hearing loss and allergic rhinitis with ADHD, using healthcare big data to provide more definitive evidence. Contrary to expectations, hearing loss demonstrated a stronger association with ADHD in girls than in boys, suggesting it could serve as an independent risk factor for ADHD.

## 1. Introduction

Attention deficit hyperactivity disorder (ADHD) is a neurodevelopmental disorder characterized by three cardinal symptoms: excessive inattention, hyperactivity, and impulsivity. ADHD is one of the most common neurodevelopmental disorders in childhood, affecting 3–8% of the pediatric population worldwide [1,2]. Given the importance of childhood development and the high prevalence of ADHD during that period, it is important to identify disorders that might cause or be associated with ADHD. Hearing loss in children could hinder their communication abilities, potentially leading to inattention and subsequent hyperactivity. Likewise, allergic rhinitis, which manifests with sneezing, rhinorrhea, and nasal obstruction, could cause inattention in children, potentially mimicking the symptoms of ADHD.

In addition to the possibility that children with hearing loss or allergic rhinitis may exhibit symptoms resembling ADHD, previous studies have suggested that these conditions might also be directly associated with ADHD, either by sharing underlying pathophysiological mechanisms or by representing overlapping spectrums of the disorder [3]. Supporting this, previous studies have revealed that hearing loss and allergic rhinitis could be associated with the prevalence of ADHD [4,5]. Some studies showed contradictory results, especially after correcting for confounding factors, such as parental smoking exposure at home [6]. Despite this, it has been observed that even after hearing restoration via cochlear implantation, patients with ADHD who were previously hearing-impaired still demonstrate a reduced capacity in auditory language and speech skills compared to those without ADHD [7]. Taken together, these findings suggest that the relationship between hearing loss or allergic rhinitis and ADHD may extend beyond a simple symptomatic overlap.

Despite these studies, there is still much room for research analyzing the associations of hearing loss and allergic rhinitis with ADHD, especially considering the influence of confounding factors. There is a particular need for studies with well-documented, large-scale population cohorts. Accordingly, the present study aimed to investigate the relationships of hearing loss and allergic rhinitis with ADHD in children by utilizing large-scale healthcare data, and to identify high-risk groups through stratified analyses by age, sex, and socioeconomic factors.

## 2. Methods

### 2.1. Data Source

South Korea has a universal national health insurance system covering the entire Korean population. We used the annual pediatric patient samples from the Korea Health Insurance Review and Assessment Service database (HIRA-PPS) from 2009 to 2018 [8]. The HIRA-PPS was constructed to include 10% of patients under the age of 20 by age/sex-stratified sampling, corresponding to approximately 1.1 million patients per year. In accordance with privacy policies, the patient samples provided by HIRA were cross-sectional and resampled on an annual basis.

The HIRA-PPS database contains a unique de-identified number (ID) for each patient, along with their age group, sex, and provider ID. Information about prescribed drugs, including the generic name, prescription date, dose, and duration, is also included. Diagnoses are coded according to the International Classification of Disease, 10th Revision (ICD-10).

### 2.2. Study Design and Study Subjects

We conducted a population-based cross-sectional study to investigate the association between the prevalence of ADHD and hearing loss or allergic rhinitis. The study population consisted of all patients aged 6 or older, and patients who were prescribed at least one prescription of either methylphenidate or atomoxetine were defined as ADHD patients. Patients who were not prescribed methylphenidate or atomoxetine during a given year were classified as non-ADHD patients.

### 2.3. Definitions of Hearing Loss and Allergic Rhinitis

Hearing loss was determined based on the existence of the diagnostic code (ICD-10: H90.x or H91.x) and an audiometry test conducted within the same year. In this study, audiometry includes auditory brainstem response threshold testing, pediatric audiometry, and pure-tone audiometry. We carried out several sensitivity analyses to evaluate the robustness of the results. These included (1) patients diagnosed with hearing loss who underwent at least two audiometry tests, (2) patients diagnosed with hearing loss in an otolaryngology department who received any audiometry tests within that same department, and (3) patients diagnosed with hearing loss in a general hospital who received any audiometry tests within that same hospital.

Patients with allergic rhinitis were identified based on the presence of a diagnostic code (ICD-10: J30.x) and the results of diagnostic tests, which included a skin test and an allergen-specific immunoglobulin E test. For a sensitivity analysis, we replicated the main analysis but restricted the definition of allergic rhinitis to those diagnosed and tested in a general hospital.

### 2.4. Statistical Analysis

The baseline characteristics, which included age group (6–9, 10–14, 15–19), sex, insurance type (health insurance or Medical Aid), and year, were summarized using frequency and proportion. In Korea, healthcare coverage is provided through the national health insurance (NHI) and the Medical Aid program. While NHI enrollees pay income-based premiums and partial copayments, Medical Aid beneficiaries represent the lowest-income groups and pay little or no medical costs, reflecting underlying socioeconomic differences. The chi-square test was used to compare the proportions of characteristics between the ADHD and non-ADHD groups. The proportion of ADHD in each subgroup was estimated by dividing the number of ADHD patients by the total number of patients in that subgroup and expressing the result as a percentage.

We estimated crude odds ratios (ORs) with 95% confidence intervals (CIs) for the association between ADHD and hearing loss or allergic rhinitis. The ORs were adjusted for the covariates described above. We explored potential effect modification through subgroup analyses by age group, sex, and year. The interactions between subgroups and hearing loss or allergic rhinitis were examined by including a cross-product term in the logistic regression model.

Data analysis was performed using SAS version 9.4 (SAS Institute, Cary, NC, USA), with the significance level set at *p* < 0.05.

### 2.5. Ethics Statement

The study protocol was exempted from review by the Institutional Review Board of Chungnam National University Sejong Hospital (IRB No. CNUSH202207005).

## 3. Results

The proportion of ADHD differed across subgroups. By sex, the prevalence was higher among boys (1.29%) than among girls (0.38%). Regarding insurance type, children covered by Medical Aid had a higher prevalence of ADHD (1.98%) compared with those covered by health insurance (0.81%). An analysis of the baseline characteristics of study participants using the chi-square test showed that boys and patients with insurance coverage through Medical Aid were more likely to have ADHD than girls or patients covered through the national health insurance system (Table 1).

Hearing loss was associated with the prevalence of ADHD (adjusted OR: 1.79; 95% CI: 1.67, 1.92). Although the point estimate for the OR for the association between allergic rhinitis and ADHD was reduced after adjustment for age, sex, insurance type, and year; nonetheless, the relationship between allergic rhinitis and ADHD remained statistically significant (Table 2). A sensitivity analysis using conditions such as more audiometry tests, diagnosis by an otolaryngologist, and diagnosis at a general hospital strengthened the associations of hearing loss and allergic rhinitis with ADHD (Table 2). Further analysis showed that patients with both hearing loss and allergic rhinitis had a higher OR than patients with either hearing loss or allergic rhinitis alone, as well as compared to patients without hearing loss or allergic rhinitis (Table 3).

Interestingly, a subgroup analysis by sex and age group showed that the association between hearing loss and ADHD was significantly stronger in girls than in boys (*p* < 0.001) For allergic rhinitis, the association with ADHD did not differ by sex (*p* for interaction = 0.465) but a significant interaction by age group was observed (*p* for interaction = 0.025). Analyses stratified by year showed consistent associations for hearing loss throughout the 10-year period (Table 4).

## 4. Discussion

In this study, we reaffirmed the link between hearing loss and allergic rhinitis in relation to ADHD, consistent with the previous literature [5,9], with particular emphasis on the stronger association of ADHD in patients affected by both conditions. Although the predisposition for ADHD in males has been well-documented [10,11], our subgroup analysis revealed that girls with hearing loss were more likely to have ADHD than boys with the same condition. Previous studies have suggested that the higher prevalence of ADHD in males may be attributable to sex-related differences in neurotransmission such as dopaminergic or noradrenergic pathways, the influence of androgen exposure on neurodevelopment, and sociocultural factors contributing to differential recognition and diagnosis of ADHD in boys versus girls. Our findings therefore underscore the strong link between hearing loss and ADHD, suggesting that hearing loss could be an independent risk factor for ADHD regardless of the baseline male predominance. Given these results, we propose that hearing rehabilitation could serve as a supplemental treatment for ADHD patients with hearing loss. This hypothesis warrants further investigation in future research.

What drew our attention was the fact that children with Medical Aid were more likely to be diagnosed with ADHD than those covered by regular NHI. It has been previously reported that low socioeconomic status, determined by factors such as parental education and marital status, is associated with ADHD [12]. Alternatively, this could simply be due to the affordability of medical expenses for those with Medical Aid. This is a topic that warrants further investigation, ideally with more detailed data.

Research on ADHD faces challenges in obtaining accurate data due to the ambiguity of diagnoses and difficulties in utilizing information. This is particularly true in Korea, where the use of data related to ADHD requires a stringent permission process since the relevant ICD-10 codes are related to a psychiatric disorder. This study stands out due to its scale, being one of the largest population studies conducted to date. Furthermore, while ADHD was previously thought to occur more frequently in men than in women, this study found that girls with hearing loss were more likely to have ADHD than boys with hearing loss. This suggests that the combined impact of sex and hearing loss on ADHD requires particular attention.

The interpretation of the current study should consider several inherent limitations. First, due to its cross-sectional nature, we cannot definitively establish a temporal relationship between hearing loss, allergic rhinitis, and ADHD. To investigate this relationship more thoroughly, future research, specifically cohort studies using a new-user design, will be necessary. Second, due to the inherent characteristics of the claims database, the lack of data on healthcare services not covered by insurance could potentially lead to inaccuracies in defining a disease comprehensively. In addition, our database did not include information on certain potential confounders, such as family history of ADHD or environmental exposures (e.g., parental smoking). Moreover, socioeconomic status was not directly measured; insurance type was used as an indirect proxy. Finally, potential inaccuracies in diagnostic codes identified within this database could not be validated by a medical chart review. However, a previous study reported that the validity of major diagnostic codes within the HIRA database is reliable [13]. Additionally, we conducted several sensitivity analyses, including diagnostic tests, department, and hospital type, to reinforce the robustness of our results.

## 5. Conclusions

Taken together, we should be more vigilant in managing children with ADHD who also have hearing loss and allergic rhinitis. These comorbid conditions require concurrent treatment, which could potentially enhance the effectiveness of ADHD management. Currently, many countries, including the Republic of Korea, have implemented newborn hearing screening programs, and preschool hearing screening is being conducted in some countries or is planned for adoption in many other countries. By leveraging these programs, clinical protocols could be designed to facilitate the early diagnosis of ADHD. Furthermore, this approach may aid in the accurate diagnosis of ADHD by reducing the possibility of overlapping diseases.

## Figures and Tables

**Table 1:** Baseline characteristics of study participants.

Characteristics		Proportion of ADHD (%)	ADHD		Non-ADHD		*p*-Value
			N	(%)	N	(%)	
Total			66,493		7,730,191		
Age group	6–9	1.00	19,032	28.62	1,877,516	24.29	<0.0001
	10–14	1.13	29,936	45.02	2,617,258	33.86	<0.0001
	15–19	0.54	17,525	26.36	3,235,417	41.85	<0.0001
Sex	Boys	1.29	52,070	78.3	3,970,961	51.37	<0.0001
	Girls	0.38	14,423	21.69	3,759,230	48.63	<0.0001
Insurance type	Health insurance	0.81	60,310	90.7	7,423,676	96.03	<0.0001
	Medical aids	1.98	6183	9.3	306,515	3.97	<0.0001
Year	2009	0.74	6471	9.73	871,333	11.27	<0.0001
	2010	0.81	7003	10.53	853,869	11.05	<0.0001
	2011	0.85	7136	10.73	827,893	10.71	0.8545
	2012	0.96	7802	11.73	806,087	10.43	<0.0001
	2013	0.89	7047	10.6	784,667	10.15	<0.0001
	2014	0.82	6392	9.61	769,792	9.96	0.0031
	2015	0.80	5992	9.01	747,208	9.67	<0.0001
	2016	0.81	5965	8.97	732,766	9.48	<0.0001
	2017	0.89	6071	9.13	675,383	8.74	0.0003
	2018	0.99	6614	9.95	661,193	8.55	<0.0001

ADHD: Attention Deficit hyperactivity disorder.

**Table 2:** Association of ADHD prevalence related to hearing loss and allergic rhinitis.

Disease	Presence of Disease	Number of ADHD (%)	OR (95% CI)	*p*-Value	Adjusted OR (95% CI)	*p*-Value
**Hearing loss**	N	65,680 (0.85%)	ref		ref	
	Y	813 (1.5%)	1.78 (1.66,1.9)	<0.001	1.79 (1.67,1.92)	<0.001
Sensitivity analysis						
Defined with at least two audiometry tests	N	66,310 (0.85%)				
	Y	183 (1.6%)	1.89 (1.63,2.19)	<0.001	1.82 (1.57,2.11)	<0.001
Diagnosed by otolaryngologist	N	65,805 (0.85%)				
	Y	688 (1.51%)	1.79 (1.66,1.94)	<0.001	1.8 (1.67,1.95)	<0.001
Diagnosed at general hospital	N	66,230 (0.85%)				
	Y	263 (1.89%)	2.25 (1.99,2.54)	<0.001	2.08 (1.84,2.36)	<0.001
**Allergic rhinitis**	N	64,672 (0.84%)				
	Y	1821 (1.4%)	1.67 (1.59,1.75)	<0.001	1.41 (1.35,1.48)	<0.001
Sensitivity analysis						
Diagnosed at general hospital	N	65,948 (0.85%)				
	Y	545 (1.55%)	1.83 (1.68,2)	<0.001	1.54 (1.41,1.68)	<0.001

ADHD: Attention deficit hyperactivity disorder, OR: odds ratio, 95% CI: 95% confidence interval.

**Table 3:** Association of ADHD prevalence related to the combination of hearing loss and allergic rhinitis.

Presence of Disease		Number of ADHD (%)	OR (95% CI)	*p*-Value	Adjusted OR (95% CI)	*p*-Value
Hearing loss	Allergic rhinitis					
N	N	63,911 (0.84%)	ref		ref	
Y	N	761 (1.47%)	1.76 (1.64,1.89)	<0.001	1.79 (1.66,1.92)	<0.001
N	Y	1769 (1.38%)	1.66 (1.58,1.74)	<0.001	1.41 (1.34,1.48)	<0.001
Y	Y	52 (2.08%)	2.53 (1.93,3.33)	<0.001	2.1 (1.59,2.76)	<0.001

ADHD: Attention deficit hyperactivity disorder, OR: odds ratio, 95% CI: 95% confidence interval.

**Table 4:** Association of ADHD prevalence related to hearing loss and allergic rhinitis according to sex, age group, and year.

Subgroups	Presence of Disease	Number of ADHD (%)	OR (95% CI)	*p*-Value	Adjusted OR (95% CI)	*p*-Value
Hearing loss *						
Boys	N	51,484 (1.29%)	ref		ref	
	Y	586 (2.07%)	1.62 (1.49,1.76)	<0.001	1.65 (1.52,1.79)	<0.001
Girls	N	14,196 (0.38%)	ref		ref	
	Y	227 (0.87%)	2.32 (2.03,2.64)	<0.001	2.26 (1.98,2.57)	<0.001
Age (6–9)	N	18,819 (1.00%)	ref		ref	
	Y	213 (1.96%)	1.98 (1.73,2.27)	<0.001	1.84 (1.6,2.11)	<0.001
Age (10–14)	N	29,601 (1.13%)	ref		ref	
	Y	335 (2.00%)	1.79 (1.61,2)	<0.001	1.74 (1.56,1.94)	<0.001
Age (15–19)	N	17,260 (0.53%)	ref		ref	
	Y	265 (0.99%)	1.86 (1.65,2.11)	<0.001	1.81 (1.6,2.04)	<0.001
Year (2009)	N	6405 (0.73%)	ref		ref	
	Y	66 (1.46%)	2.01 (1.58,2.57)	<0.0001	2.00 (1.57,2.56)	<0.0001
Year (2010)	N	6935 (0.81%)	ref		ref	
	Y	68 (1.38%)	1.71 (1.34,2.17)	<0.0001	1.68 (1.32,2.14)	<0.0001
Year (2011)	N	7040 (0.85%)	ref		ref	
	Y	96 (1.73%)	2.06 (1.68,2.53)	<0.0001	2.1 (1.71,2.58)	<0.0001
Year (2012)	N	7709 (0.95%)	ref		ref	
	Y	93 (1.74%)	1.85 (1.5,2.27)	<0.0001	1.84 (1.49,2.26)	<0.0001
Year (2013)	N	6958 (0.89%)	ref		ref	
	Y	89 (1.6%)	1.83 (1.48,2.26)	<0.0001	1.87 (1.51,2.31)	<0.0001
Year (2014)	N	6316 (0.82%)	ref		ref	
	Y	76 (1.38%)	1.7 (1.35,2.13)	<0.0001	1.71 (1.36,2.15)	<0.0001
Year (2015)	N	5921 (0.79%)	ref		ref	
	Y	71 (1.25%)	1.59 (1.26,2.01)	0.0001	1.59 (1.25,2.01)	0.0001
Year (2016)	N	5882 (0.80%)	ref		ref	
	Y	83 (1.35%)	1.69 (1.36,2.1)	<0.0001	1.7 (1.37,2.12)	<0.0001
Year (2017)	N	5993 (0.89%)	ref		ref	
	Y	78 (1.36%)	1.54 (1.23,1.93)	0.0002	1.6 (1.28,2)	<0.0001
Year (2018)	N	6521 (0.98%)	ref		ref	
	Y	93 (1.71%)	1.75 (1.42,2.15)	<0.0001	1.82 (1.48,2.24)	<0.0001
**Allergic rhinitis ^†^**						
Boys	N	50,570 (1.28%)			ref	
	Y	1500 (1.93%)	1.52 (1.44,1.6)	<0.001	1.39 (1.32,1.46)	<0.001
Girls	N	14,102 (0.38%)				
	Y	321 (0.61%)	1.61 (1.44,1.8)	<0.001	1.5 (1.34,1.67)	<0.001
Age (6–9)	N	18,364 (0.99%)				
	Y	668 (1.42%)	1.43 (1.33,1.55)	<0.001	1.29 (1.2,1.4)	<0.001
Age (10–14)	N	29,144 (1.12%)				
	Y	792 (1.85%)	1.66 (1.55,1.79)	<0.001	1.49 (1.39,1.6)	<0.001
Age (15–19)	N	17,164 (0.53%)				
	Y	361 (0.89%)	1.68 (1.51,1.86)	<0.001	1.53 (1.37,1.7)	<0.001
Year (2009)	N	6296 (0.73%)				
	Y	173 (1.52%)	2.11 (1.81,2.45)	<0.0001	1.74 (1.5,2.03)	<0.0001
Year (2010)	N	6809 (0.80%)				
	Y	194 (1.58%)	1.98 (1.72,2.29)	<0.0001	1.69 (1.47,1.96)	<0.0001
Year (2011)	N	6954 (0.85%)				
	Y	182 (1.43%)	1.7 (1.47,1.97)	<0.0001	1.42 (1.22,1.65)	<0.0001
Year (2012)	N	7623 (0.95%)				
	Y	179 (1.44%)	1.52 (1.31,1.76)	0.0008	1.28 (1.1,1.48)	0.0015
Year (2013)	N	6863 (0.88%)				
	Y	184 (1.41%)	1.61 (1.39,1.86)	<0.0001	1.38 (1.19,1.6)	<0.0001
Year (2014)	N	6210 (0.81%)				
	Y	182 (1.32%)	1.63 (1.4,1.89)	<0.0001	1.44 (1.24,1.67)	<0.0001
Year (2015)	N	5833 (0.79%)				
	Y	159 (1.20%)	1.53 (1.3,1.79)	<0.0001	1.35 (1.15,1.58)	0.0002
Year (2016)	N	5807 (0.80%)				
	Y	158 (1.13%)	1.42 (1.21,1.67)	<0.0001	1.22 (1.04,1.43)	0.0159
Year (2017)	N	58,852 (0.88%)				
	Y	189 (1.42%)	1.62 (1.4,1.87)	<0.0001	1.35 (1.16,1.56)	<0.0001
Year (2018)	N	6393 (0.98%)				
	Y	221 (1.56%)	1.61 (1.41,1.84)	<0.0001	1.37 (1.2,1.57)	<0.0001

ADHD: Attention deficit hyperactivity disorder, OR: odds ratio, 95% CI: 95% confidence interval. * *p* for interaction: age (<0.001), sex (0.702), year (0.726). **^†^** *p* for interaction: age (0.465), sex (0.025), year (0.009).

## Data Availability

The original contributions presented in this study are included in the article. Further inquiries can be directed to the corresponding author.
